# Local Alcohol Policy Implementation in Scotland: Understanding the Role of Accountability within Licensing

**DOI:** 10.3390/ijerph16111880

**Published:** 2019-05-28

**Authors:** Alex Wright

**Affiliations:** Global Health Policy Unit, Social Policy, School of Social and Political Science, University of Edinburgh, Edinburgh, EH8 9LD, UK; alex.wright@ed.ac.uk; Tel.: +44-(0)-131-6504147

**Keywords:** alcohol policy, licensing, accountability, policy implementation, Scotland

## Abstract

Scotland has been ambitious in its policy and legislative efforts to tackle alcohol-related harm, efforts which include the innovative feature of a ‘public health objective’ within local alcohol licensing. However, the persistence of alcohol-related harms and inequalities requires further examination of both the overarching Scottish alcohol strategy and its specific implementation. A qualitative case study was undertaken to explore how alcohol policy is implemented locally in Scotland, with data generated from (i) documentary analysis of 12 relevant policies, legislation, and guidance documents; and (ii) a thematic analysis of semi-structured interviews with 54 alcohol policy implementers in three Scottish localities and nine national-level stakeholders. The data suggest there is a tension between the intentions of licensing legislation and the way it is enacted in practice, and that accountability emerges as an important factor for understanding why this occurs. In particular, there are a lack of accountability mechanisms acting upon Scottish Licensing Boards to ensure they contribute to the public health goals of the Scottish alcohol strategy. From a public health perspective, this has perpetuated a system in which Licensing Boards continue to act with autonomy from the rest of the alcohol policy implementation system, creating a challenge to the achievement of public health goals. Alcohol policy in Scotland is likely to fall short of intended goals as long as the tension between licensing legislation and enacted licensing practices remains.

## 1. Introduction

This study examines the ‘how and why’ of Scottish alcohol policy implementation. A key finding which emerged from the study was the importance of accountability, particularly within the context of Scotland’s alcohol licensing regime. This article focuses on explaining how accountability mechanisms within Scotland’s licensing regime can present challenges to the effective implementation of alcohol policy. This is important because limited empirical research has examined how processes of accountability within alcohol licensing influence Scottish alcohol policy implementation and the related pursuit of public health goals. This study investigates this problem by undertaking a qualitative case study, using documentary analysis of national policy, legislation and guidance, and interview data with national and local alcohol policy implementation stakeholders.

In Scotland, alcohol misuse is an important public health policy problem; high levels of alcohol consumption have contributed to patterns of health inequalities and led to correspondingly high rates of alcohol-related harms and costs [1]. To address these issues, the Scottish Government developed policy and legislation to prevent and address alcohol-related harms. The government’s central policy, *Changing Scotland’s Relationship with Alcohol: A Framework for Action*, was published in 2009 [2]. It outlines a ‘whole population approach’ as a key feature [1] and has been lauded as being ‘evidence-informed’ [3]. An additional innovative feature of the government’s overall approach has been the inclusion of a ‘public health objective’ within licensing legislation, namely the *Licensing (Scotland) Act 2005*.

Extensive evaluations of population health outcomes have also been undertaken in relation to Scottish alcohol policy [4]. These have demonstrated, for example, that while alcohol-related death rates in Scotland peaked in the mid-2000s and then began to fall, this trend has stalled and, since 2012, rates have been increasing again [5,6]. Additionally, while inequalities in alcohol-related deaths in Scotland have narrowed over time, alcohol-related mortality rates remain more than eight times higher in the most deprived areas compared with least deprived areas [5]. It is apparent that while some progress has been made in tackling alcohol-related harm, there is a continued need for the effective implementation of the evidence-informed measures embedded in Scottish alcohol policy.

In light of this, there is a need to understand the challenges and facilitators of Scottish alcohol policy implementation—a contribution this article seeks to make, with a specific focus on alcohol licensing and the emergent theme of accountability. This article is timely given recent policy developments within Scottish Government which will need to be implemented effectively. This includes the publication in late 2018 of new Scottish alcohol policy [7,8] to improve health through the prevention of alcohol- and drug-related harm and a revised draft of the *Guidance for Licensing Boards* which, at time of writing, is out for consultation [9].

### Scottish Alcohol Licensing: Policy Context, Existing Research and Accountability

While the current Scottish approach to alcohol policy has been led by a Scottish National Party (SNP) government, there has been notable cross-party support for an alcohol strategy generally, providing a supportive political context for this issue. The context is complicated, however, by the Scottish Parliament and Government’s constrained capacity to act only on elements of alcohol regulation which have been devolved to them by the UK Government [10]. Additionally, the past few years have seen substantive policy and political energy at a national level dedicated to the development and enactment of legislation for minimum unit pricing (MUP) of alcohol [11]. The Scottish Parliament passed MUP legislation in 2012, but was then forced to fight a protracted legal battle in European and UK courts against a challenge brought by the Scotch Whisky Association [12]. The government’s case was eventually successful, and MUP was implemented in May 2018; however, in the meantime, other local processes of alcohol policy implementation were facing their own challenges. Within this context, this study was concerned with the ongoing implementation of the range of other alcohol policy measures in the Scottish alcohol strategy, including localized decision-making (e.g., licensing) by locally elected representatives and other stakeholders such as Alcohol and Drug Partnerships (ADPs).

Alcohol licensing is a key competency devolved to the Scottish Parliament from the UK Government in Westminster. The *Licensing (Scotland) Act 2005* (referred to here as LA 2005), helps to structure the legislative and regulatory framework for licensing and makes provisions for the regulation of the sale of alcohol and the premises on which alcohol is sold. LA 2005 gives local Licensing Boards (LBs) the responsibility for granting or rejecting alcohol licenses and thus helps to determine the availability of alcohol in local areas. The membership of these LBs is constituted of locally elected councillors.

Critically, LA 2005 contains five ‘licensing objectives’, and requires LBs to be concerned with each: (i) Preventing crime and disorder; (ii) securing public safety; (iii) preventing public nuisance; (iv) protecting and improving public health; and (v) protecting children from harm. There are intersections across all five objectives, and all potentially run counter to economic interests invested in alcohol sales. Of these, however, the implementation of the ‘public health objective’ is most relevant for this article, and it was primarily in relation to this objective and the policy context surrounding it that accountability emerged as an explanatory factor.

In the UK, Scotland is unique for including the protection of public health as a statutory objective in its licensing legislation [3,13]. Indeed, the Scottish alcohol strategy identifies alcohol licensing as a key component of their ‘whole-population approach’ to combating alcohol-related harm, and it identifies licensing stakeholders as having a key role in helping to achieve the strategy’s public health goals.

The public health objective gives LBs a duty to assess the number and density of licensed premises in their area—a key measure if one is concerned with the availability of alcohol. This is operationalized in the concept of ‘overprovision’, which refers to an “assessment that there are too many licensed premises in a particular locality either in terms of the number of premises, the capacity of premises, the type of premises, or the size of a display area” (p. vi, [14]).By distinguishing a given area as overprovided for, LBs have policy grounds to refuse new license applications in this area.

In Scotland, the assessment of whether overprovision exists happens during the development of each LB’s ‘Licensing Policy Statement’ which “sets out the general approach a Licensing Board will take to regulating the sale of alcohol and licensed premises in its area” (p. 2, [15]). Importantly, the Statement must include a declaration of how each LB will progress towards each of the five licensing objectives, and LBs must make their licensing decisions with consideration to their Policy Statement [15].

In terms of policy practice, alcohol licensing in Scotland occurs in local government, where local councils and their respective elected councillors have a certain level of autonomy from the Scottish Government. This is grounded in a Concordat signed by the Scottish Government in 2007 with the Convention of Scottish Local Authorities (COSLA), which removed certain controls that the government had over councils [16]. This has implications for LB members’ role in alcohol policy implementation, since their status as councillors means they are not automatically obligated to follow Scottish Government-identified priorities (e.g., to commit to a whole-population approach to tackling alcohol-related harm). However, this autonomy is complicated by the government’s parallel implementation of the National Performance Framework, an instrument first implemented in 2007 (and recently revised in 2018) which defines the government’s ‘purpose’ and overarching goals [17]. As part of the aforementioned Concordat, local governments have to identify their local priorities through community planning and demonstrate how these contribute to the National Performance Framework [16].

A range of organisations are stakeholders in Scottish alcohol policy enactment; these include the Scottish Parliament and Scottish Government, national governmental organisations concerned with health, and local entities such as local authorities, local partnerships, and local communities. The specific focus in this article is the relationships of LBs with both the Scottish Government and local Alcohol and Drug Partnerships (ADPs). The membership and responsibilities of each are shown in Table 1 below.

Research on the interplay between local alcohol availability and health has proliferated in recent years in the UK, and this research demonstrates both that availability is associated with population harm [19,20,21] and that policies to regulate availability can have a positive impact on population health [22]. For example, Richardson et al. (2015) have demonstrated that in Scotland, a higher alcohol outlet density in a given neighbourhood is associated with higher alcohol-related hospitalisations and deaths [20], while research from the broader UK context suggests stricter licensing enforcement to regulate alcohol availability may have a positive effect on alcohol-related hospitalisations [23]. Note, however, that existing research on outlet density is not definitive in providing policy decision-makers with density thresholds that should not be exceeded. Therefore, licensing decisions informed by this work will remain interpretative. Further, while criticisms of this area of literature have noted limitations in terms of methodological approaches and scope [24], as well as the currently inability to demonstrate causality [25], existing research indicates the importance of local licensing decision-making on population health outcomes.

In addition to the association between alcohol availability and harm, research has also studied the licensing policy context [13,26] and different aspects of licensing processes. In the Scottish context, an in-depth evaluation of the implementation of LA 2005, an evaluation which found a number of key challenges that prevented effective implementation of this legislation [14]. These included a lack of updated implementation guidance and the inconsistent manner in which national and local data was being collected [14].

An additional key issue evident in the literature has been the interplay of evidence used within licensing processes. In particular, research has suggested that public health evidence has limited impact on licensing decision-making, and LB members more often rely on their own values and beliefs, or anecdotes from their constituencies, to inform their decisions [27]. Further, that while public health interviewees in this study perceived themselves to be approaching their work with a ‘whole-population approach,’ the same perspective was not always adopted by other licensing stakeholders (i.e., LB members) [27]. These results have highlighted a possible tension between the perspectives, goals, and priorities among different licensing stakeholders.

While the above research contributes to an understanding of licensing processes, there remains a limited body of empirical research in the UK and Scottish contexts reporting exactly how key stakeholders are being held accountable for their role in effectively implementing licensing policy or how the accountability regime(s) surrounding licensing influence alcohol policy implementation. Indeed, recent work by Fitzgerald and colleagues [28] is unique in its explicit inclusion of accountability as a theme in their analysis of Scottish licensing. Their research reported (i) a lack of mechanisms available to influence the councillors who were members of local LBs and (ii) that LB convenors and licensing clerks had the power to shape a given LB’s attitude towards public health. Further, that the latter situation sometimes resulted in challenges to local public health progress and variations across local areas in terms of how the public health objective was perceived and implemented. As will be shown in the results and discussion sections, this article helps to reaffirm and build upon those authors’ work.

Overall, however, specific in-depth inquiries into accountability in alcohol policy implementation, remain limited, and findings are not linked to existing accountability literature, a substantive area of research from which theoretical and empirical lessons may be drawn. While literature on alcohol policy implementation studies have sometimes discussed accountability-related issues (e.g., in relation to power in licensing processes [28], the importance of clearly establishing responsibility for particular interventions [29], or policy stakeholders’ compliance with and navigation of relevant alcohol legislation [14,30]), a notable gap in published research exists which draws explicitly upon lessons from accountability scholarship to empirically examine alcohol policy implementation processes. Given the emergence in this research of accountability as an explanatory factor influencing implementation of Scottish licensing policy, this article seeks to contribute understanding to this gap.

Indeed, the issue of accountability, a concept used extensively (albeit often somewhat opaquely) in public discourse, is somewhat rarely empirically examined within broader health policy implementation research. In a literature review of empirical health policy implementation studies conducted for the Scottish Parliament’s Information Centre [31], only a small number of empirical articles explicitly linked accountability and health policy implementation processes (e.g., Kelly et al. [32] and O’Toole et al. [33]). This is despite authors within public policy and implementation literature identifying accountability as being fundamental to policy implementation. For example, Jan-Erik Lane [34] has written that the implementation gap between policy expectations and outcomes is inherently related to accountability.

The current paper is situated in the context of the existing regulatory and accountability framework for licensing stakeholders—LBs in particular. Table 2 outlines the relevant provisions regarding accountability of LBs as stated in existing legislation.

In relation to this legal framework and the policy context described above, this article seeks to address the existing knowledge gap at the intersection of Scottish alcohol policy implementation, licensing, and accountability. It asks whether the ways LBs are held accountable function to support implementation processes and corresponding public health goals in the context of Scotland’s national alcohol strategy. Towards this aim, this article examines how LBs, as administrative and quasi-judicial entities that exist beyond the traditional health arena, can have important impacts on implementation processes and subsequent health policy outcomes.

## 2. Methods

This research was conducted as part of a doctoral research project which aimed to generate understanding about how and why Scotland’s national alcohol strategy was implemented in local areas. This overarching study aim lent itself to using a qualitative case study approach to attain a rich, ‘thick’ description of processes within Scottish alcohol policy implementation. Ethical approval for this study was granted by the School of Social and Political Science at the University of Edinburgh.

Three purposely selected local authority areas (referred to here as ‘local areas’) across Scotland served as the study sites in order to explore similarities and differences in alcohol policy implementation across different settings. The three local areas were selected using an established sampling framework from Miles and Huberman [35], which incorporates theoretical and pragmatic considerations such as whether the sample plan is feasible, ethical, and has the potential to generate rich information. Within the framework, a researcher also has the flexibility to account for other relevant considerations; for example, in this study, sites were selected to attain a diversity of urban and rural locations. Ultimately, one urban (LA1), one mixed urban-rural (LA2), and one rural (LA3) site agreed to participate in this study, and ethical approval was attained from the relevant authority in each prior to data collection.

Data were generated from the analysis of policy documents and semi-structured interviews. The approach to documentary analysis followed Mason in viewing the content of key policy documents as constructed representations of a formal decision (or set of decisions) taken by those with authority in the Scottish alcohol policy system [36], as well as providing insights into the values underlying these policy decisions [37]. These insights were needed to help to understand the representations of policy implementation in the documents (in terms of both process and outcomes) and whether they contained information about the policy context and about the roles and expectations of alcohol policy implementers (i.e., LBs or ADPs). In contrast, the use of interviews allowed an understanding of the perceptions of alcohol policy implementers themselves regarding how they undertook policy implementation practice. These data were generated from semi-structured interviews with national-level alcohol policy stakeholders and local alcohol policy implementers.

Overall, a documentary analysis of 12 relevant national policies, legislation, and reports was undertaken. The analysis was guided by Walt and Gilson’s [38] Triangle Framework for health policy analysis, which focuses on actors, context, process, and content. While local policy documents (e.g., LBs’ Statement of Licensing Policy) were read by the author, they were not formally reported on within the study to preserve the anonymity of each participating local area.

For the interviews, national interviewees (n = 9) were recruited if they were currently involved in the development and delivery of alcohol policy and/or legislation in Scotland or if they had been involved in the development of the 2009 *Framework for Action*. This included a range of representations across public and third sector organisations. Local interviewees were recruited from the three selected local areas’ respective LBs (n = 8) and ADPs (n = 46) (Table 3).

ADPs are local, multi-sectoral partnerships tasked by the Scottish Government with carrying out alcohol policy implementation; their membership includes, for example, representatives from the local health board, police, community justice, social work, education, and the third sector [39]. There is a total of 30 ADPs across Scotland, which are usually matched geographically with the boundaries of their respective local authority area. ADPs do not have a formal relationship with LBs; however, certain ADP members (specifically police and health) are ‘statutory consultees’ to the LB, meaning they have the right to be informed when a license application is submitted and to lodge an objection to that application if they wish. They do not, however, have a role in the decision about whether to grant a licence. Further, ADPs and their members work within a context of ‘community planning’—mandated collaborative working of public services and communities to design and deliver services—established in Scotland in 2003 [40,41]. In each local area, a ‘Community Planning Partnership’ (CPP) is responsible for determining local policy priorities—organizations such as ADPs report to the CPP, while LBs do not.

All interviewees were recruited directly via email, and interviewing took place between December 2015–January 2017. Interviews ranged from 40 minutes to two hours in length, and each was audio recorded with the interviewee’s consent. The interview schedule was developed on the basis of literature on alcohol policy and policy implementation. Interviews were analysed thematically [42] in NVivo 10 [43] using a combination of deductive and inductive approaches [44,45]. For the deductive coding, provisional codes were created based on the study’s research questions, the author’s understandings of relevant policy implementation literature, and recollection of the interviews themselves. For the inductive coding, new codes were generated a posteriori from the data as new themes, topics, or concepts were identified within the transcripts. Analysis of interviews and documents occurred in parallel.

Data generated around the theme of accountability provided rich, meaningful information about the actions of alcohol policy implementers. While multiple types of formal and informal accountability emerged from the data, this article reports on the ‘public-administrative’ accountability of licensing decision-makers, a type of accountability which examines vertical or hierarchical accountability relationships—with regards to political or legal interactions, for example [46]. Thus, the analysis investigated accountability mechanisms and arrangements in which one organisation is held accountable by another who is ‘higher’ in the formal governance hierarchy.

The combination of documentary data and interview data provided both official government documentation of stated intentions with regards to the alcohol strategy and first-hand accounts of alcohol policy implementation practice, respectively. The multiple sources of data were integrated to find where there was alignment or tensions between formally written or stated expectations of alcohol policy implementation and what was enacted in practice. As noted, following the emergence of accountability as an important theme in the broader research project, this article is concerned with potential differences between expectations and practice through the lens of accountability within alcohol licensing. In particular, the focus here is whether the licensing accountability regime as structured and enacted presents challenges or enables Scottish alcohol policy implementation.

## 3. Results

### 3.1. Perspectives on the Licensing Objectives and Importance of Accountability

Within interviews, LB members and other licensing stakeholders discussed the licensing objectives, contrasting the public health objective with the priority afforded to the other objectives. In particular, it was reported that the public health objective had not yet led to major changes in how licensing stakeholders operated in relation to public health concerns:

“I think [the public health objective] has always been the poor cousin of the five licensing objectives. It’s a difficult one for the Board to deal with, because an application where there are issues of disorder, or public nuisance, noise, whatever, in an immediate area, that’s quite clear… and the police might provide evidence to that effect…they can tie in a refusal with the kind of disorder and public nuisance licensing objectives. Public health has always been more of a difficult one for the Board… the Board doesn’t really see favour with overprovision as a concept”

(LA1, job title withheld)

One of the key explanations for these challenges associated with implementing the public health objective was the accountability arrangements surrounding LBs. For example, one ADP member reported on frustrations being raised across organisations involved in local alcohol policy implementation, in relation to the lack of accountability surrounding LBs:

“[We were]…working with the police and the NHS, to try to influence the Licensing Board, nothing really happening, and then that frustration coming back at the Community Planning Partnership level because they can’t influence it either. So yeah, Licensing Boards, because of legislation, are sitting out here doing as they please without any accountability.”

(LA1, ADP Member)

### 3.2. Legal Accountability of Licensing Boards

Legal accountability is a type of accountability in which expectations are based on legal norms and rules and are enforced by legal bodies (e.g., courts) [47]. As previously noted in Table 2, multiple pieces of legislation determine the legal obligations of LBs. However, while this legislation provides the legal accountability framework for LBs, the data suggest LBs have certain flexibility in enacting this framework, which has implications for how they contribute to the public health-related aspects of the legislation and Scotland’s whole-population approach overall.

An example of this is in their development of overprovision statements. LA 2005 has the accompanying *Guidance for Licensing Boards and Local Authorities*, which notes the duty of LBs to assess overprovision in their area; it also notes that LBs were meant to make an “accurate assessment of overprovision” [48]. However, the *Guidance* does not specify what an ‘accurate’ assessment was to entail. The only existing legal stipulation is that LBs must demonstrate they have considered the number and capacity of licensed premises in the area and have conducted certain mandatory consultations with, for example, statutory services and the public. However, the number and capacity at which an area should be designated as overprovided is determined by the Board itself—there is not a uniform threshold against which areas are measured, nor is there a national ‘example’ or template overprovision statement from which LBs can draw [49]. Accordingly, LBs act autonomously to interpret availability-related evidence and establish local thresholds for overprovision as they see fit.

For example, ADP members reported their frustration in attempting to inform LB decisions regarding their Policy Statement and overprovision:

“You can put all the evidence and science in front of them that you like showing that link doing all you know, setting out the particular concerns you’ve got for parts of [LA1], chances are they’re not going to take it on board.”

(LA1, ADP Member)

This ADP member described working with other local licensing stakeholders to provide an evidence-informed report to the LB, which recommended certain local areas be labelled ‘overprovided,’ noting that this was unsuccessful given the autonomy LB members had to make the final decision. The existence of LB decision-making autonomy is supported by evidence of the variation in Licensing Policy Statements across Scotland [15].

The interview data also illustrated that LBs recognised and consciously used their existing discretion within the legislative framework. The quotation below is an example which illustrates how Board members spoke about their consideration of the relevant legislation, in which an element of discretion is also evident:

“The licensing laws…they keep everybody tight on what way we should be going or what we can do or can’t do or if we’ve got leeway in a certain place.”

(LA3, LB Member)

This statement was made in the context of explaining how the LB had made a decision to change a local policy about licensed premise curfews. S/he explained that these types of decisions must be seen by the LB’s legal team to ensure that local decisions are aligned with, or ‘tight on,’ national legislation, but that in this instance, the LB had the discretion, or ‘leeway,’ to make a decision regarding curfews. This demonstrates that interpretation and flexibility is present in the process of implementing the legislation. Evidence shows there are also specific personnel who help LBs to navigate the licensing legislation—legally qualified clerks which are mentioned throughout LA 2005 [50]. A similar, institutional support mechanism does not exist for other aspects of the Boards’ public-administrative accountability, further indicating the dominance of legal accountability in the LB’s accountability arrangements.

The flexibility within the licensing legislative framework seems to have been intentional on the part of the government. For example, in the cover letter of the associated *Guidance for Licensing Boards*, Gary Cox, Head of the Licensing Team states:

“I would like to stress that Boards will have the flexibility to operate and take decisions in light of their particular circumstances…That is a fundamental principle of the [Licensing] Act, and it is important to maintain it. The guidance does not seek to instruct Boards exactly how to make the Act work.”

Guidance for Licensing Boards (2007) [48]

This purposeful flexibility gives LBs a significant amount of autonomy to interpret and implement the legislation. The findings from this research suggest that this flexibility has contributed to the limited implementation of the public health objective. For example, as will be discussed below, LB members often choose to prioritize local economic concerns (which are not enshrined in a licensing objective), presenting a potential challenge to the pursuit of public health goals.

When asked what sanctions would be applied if LBs were to diverge from their legal responsibilities, interview responses suggested that the primary fear was licensing decisions being appealed in court by the licence applicant, and that the cost of this would be significant.

“Well the Board’s accountable. I mean, it’s accountable by the reason that if it makes the wrong decisions, it ends up in court…and costs the council, you know, £50,000, £70,000, £100,000 in the court case.”

(LA3, LB Member)

By ‘wrong decision’, this interviewee is referring to a decision which can be legally challenged because it appears to be in error of the law or against the LB’s Policy Statement. This legal challenge is the clearest mechanism of LB accountability (i.e., an LB may face consequences for their decisions) that was generated from the research findings. In this instance, a licence applicant (e.g., supermarket chain, or restaurant) or a licence holder (whose licence has been varied, suspended, or revoked) can appeal to the Sheriff Court, a civil court in Scotland [51]. In this system, LBs are held to account by the court as an organisation. This type of legal accountability is an important accountability mechanism which can prevent the abuse of public powers and which operates independently from the political process [52].

If a licence applicant or holder wishes to trigger an appeal of an LB decision, they can do so within 21 days [51]. In contrast, ‘objectors’ to an application (who may, for example, be a statutory consultee like the police/NHS or members of the public) are not able to appeal a decision [53]. Therefore, the same routes for triggering legal accountability mechanisms do not exist for objectors. There is therefore an inbuilt imbalance between the powers of licence applicants and alcohol policy implementers. Additionally, given the costs associated with mounting legal challenges, this system favours those with greater financial resources, and it seemed evident, from the interview data, that this informed members sense of where challenges were likely to originate from (and where not). For example:

“I think the Board…has a lot of responsibility and a lot of authority that’s pretty much unchallenged unless you can afford to go to a Sheriff to overturn a decision. I mean if…we refuse alcohol in a BP [formerly ‘British Petroleum Company’] service station, BP will take us to court…But, small retailers won’t, it’s just not worth it”

(LA1, LB Member)

### 3.3. Flexibility Permits Licensing Board Prioritisation of Economic Considerations Over Public Health

The flexibility of LBs to interpret legislation discussed above was also evident in interview data which discussed local economic concerns. The way economic considerations and public health can come into conflict within Scottish alcohol policy has been noted in existing peer-reviewed literature [27], which this research complements by approaching the issue from an accountability perspective.

Multiple LB members discussed the need for licensed premises to contribute to employment and the economy, despite the absence of the economy being a consideration in the LA 2005 licensing objectives. For example:

“A lot of places…they need their licensing outlets…it’s job provision. It’s like having a factory, you know…so that’s the way you’ve got to look at it”

(LA3, LB Member)

These types of responses indicate that Board members’ interpretations of the licensing legislation and objectives is flexible enough that they can take into consideration the (local) economy, even if this leads them to make decisions that go against the licensing objectives and increases the availability of alcohol in a given area. Other Board member interviewees displayed similar concerns: That the anticipated money and jobs that licensed premises might provide were considerations when they decided on a licensing application. However, LBs have a formal, legislative-determined responsibility for progress towards the public health objective and no formal responsibility for being concerned with the economy, and yet their concerns typically seem to be much more focused on the latter.

The tension between LB prioritisation of the economy over public health was explicitly discussed by one LB member. However, this was a minority voice among LB interviewees.

“I’m aware that other Board members have conversations about the economic impact of their decisions. Now obviously under the [Licensing] Act they’re not supposed to take that into account at all, and I certainly try not to when I’m making decisions, but I know that other Board members do, and I’ve been told, for example, in the members’ lounge, ‘well, if that supermarket wasn’t going to setup there then it would just be another empty unit for years to come and they’re providing jobs anyway, so why on earth are you standing in their way?’ I think that’s a somewhat short-sighted approach and doesn’t take into account a fair bit of evidence that suggests that adding another off-licence in an area that’s already over provided for is just likely to make problems with alcohol and over consumption of alcohol worse.”

(LA1, LB Member)

Recognition of this was also evident in national level interviews:

“[Licensing Boards] sit outside that local accountability. And I suppose the tension between the licensing objectives and what they see as their economic objective now licensing doesn’t have an economic objective that it has for five licensing objectives, but they still see themselves as having an economic objective, and that probably provides quite a lot of tension.”

(National Level 3)

This quotation suggests that some LB members have adopted a sense of accountability for pursuing economic objectives relating to perceived local needs and that this is felt more strongly than their obligation towards the licensing objectives, despite the legal framework attached to the objectives. It also suggests that this approach will be maintained as long as LBs are excluded from other local accountability structures (e.g., reporting to the local council). The problem with this tension is that, from the perspective of other local implementers, it challenges local alcohol policy implementation:

“Unfortunately a lot of our objections haven’t met with much success, and the Board have granted applications that we’ve objected to…sometimes [licence applicants’] lawyers quote economic reasons, employment, and all of those reasons, whilst it might be a factor in the decision-making, it shouldn’t really be because they should be basing decisions on the licensing objectives and the legislation.”

(LA1, Police Representative)

The police are a statutory consultee on every license application and thus can file formal objections to any application. (Note, both police and a local area’s Health Board are individual statutory consultees to the LB and, in this capacity, are permitted to lodge objections to applications). In the above, a police representative suggests that LB members have made licensing decisions based on information surrounding the economy or employment. These are not only unsupported legal grounds for licensing decision-making but may also come into direct tension with the licensing objectives and legislation (and existing public health research). Further, this person seems to suggest that these economic reasons are used by the LB to overrule local statutory objections to applications. This means that economic considerations may be a threat to this mechanism by which local statutory actors can attempt to influence the restriction of alcohol licences. This is despite the existing *Guidance for Licensing Boards* stating,

“Commercial considerations are irrelevant to a policy which is designed to protect the wider public interest”

Guidance for Licensing Boards (2007) [48]

While formal guidance for policy action separates commercial considerations and public interest, it appears that the level of flexibility allowed to LBs within their legal accountability arrangements has created space for economic considerations to push aside public health concerns.

### 3.4. Lack of Accountability to Scottish Government

Data from both documents and interviews generated an understanding regarding LB accountability in relation to the Scottish Government. In the government’s *Framework for Action*, LBs are mentioned multiple times as contributors to reducing alcohol-related harm in Scotland, even more frequently than ADPs [2]. However, the language around holding LBs to account for these contributions is restrained. For example, the *Framework* states, “we will *encourage* local Licensing Boards to develop local solutions to address local problems.” (p. 14, [2], emphasis added). This inscribed language suggests that the government recognizes and perpetuates the autonomy of LBs, indicating in its communications that it must request, not demand, their cooperation in the whole-population approach to reducing alcohol-related harm. National level interview data also suggested the government was clear that LBs were not accountable to them:

“Licensing Boards aren’t accountable to Scottish Government. So we were not performance managing this across the whole system.”

(National Level 4)

This perception seems to have been clearly communicated to local level—nearly all LB members interviewed indicated they were not accountable to the Scottish Government. Further, LB interviewees reported that they did not perceive the Scottish Government to be actively monitoring their decisions or actions.

What is notable here is the tension between the Scottish Government’s role in defining Scotland’s approach to tackling alcohol related harm and the inability to hold a key set of organisations to account for contributing to this effort. If the Scottish Government is providing the mandate to pursue public health goals through the implementation of their alcohol strategy but cannot hold LBs to account for their role in this, then LBs will continue to be relatively free to prioritise other concerns. As an example, the quotation below highlights that the purpose of a national policy is nullified if LBs can simply ignore it.

“You can have a national policy up here, but if the Board’s just ignoring it, I’m not suggesting the Board is ignoring it, but we might sometimes ignore it, what will you do about it? You know I don’t think there’s any accountability to the Scottish Government to say, ‘So you can sit and make a big document to sit on the shelf all you want, but we’ll just ignore it.’ And what are you going to do about it. So, I’m not sure there’s any point in having a national policy document if Licensing Boards can just make their own minds up.”

(LA1, LB Member)

Again, the tension between LB members’ roles as Board members and as local councillors is evident. The Scottish Government has implemented legislation which places councillors on quasi-judicial, administrative boards but has not developed a corresponding system to hold them accountable for their actions on it. Further, it will be difficult for the Scottish Government to enact accountability over councillors because it risks undermining local democracy. These results demonstrate that this particular gap in LB accountability constitutes a barrier to full alcohol policy implementation because it means LBs’ responsibility for contributing to public health goals are not enforced by existing governance structures.

Additionally, this lack of LB accountability to the Scottish Government further distinguishes LBs from other alcohol policy implementers such as ADPs. For example, the *Framework for Action* uses stronger language surrounding the responsibilities of ADPs, including phrases such as “we *expect* decisions…” [2] (emphasis added) when discussing the roles of ADPs. This suggests the Scottish Government feels differently (and less strongly) about the accountability of LBs to the government in comparison with ADPs. Additional interview data (beyond the scope of this article and will be reported separately) also demonstrated that ADPs perceive themselves to be accountable to the Scottish Government. Overall, it appears there are important differences between LBs and other alcohol policy implementers (i.e., ADPs) in terms of their accountability relationships with the Scottish Government, and that these differences seem to be led by national level. This is important because it reveals the variation in accountability arrangements across different alcohol policy implementation stakeholders, which may have implications for implementation processes.

### 3.5. Critique of Licensing Board Accountability

Within the interview data, critiques of the current licensing accountability regime were evident. Of greatest interest was that certain LB members were critical of their own accountability arrangements and advocated for greater consideration of public health outcomes by the LB. For example, two LB members from LA1 were particularly critical of current LB governance and accountability throughout their interviews. The quotation below discusses an LB member’s issues with how their Policy Statement was developed:

“[The Licensing Board]’s not very accountable and it’s not very transparent…//… I have no great problem with people making decisions that I disagree with, but I do think they should be accountable for those decisions and at the moment they aren’t entirely”

(LA1, LB Member)

This quotation speaks to the issue of LB governance and transparency as it relates to accountability. It also raises the issue that if an individual or organisation wanted to hold the LB to account for the final content it puts in its Policy Statement, it would be difficult for one to do so. The quotation below demonstrates this:

“We pretty much make our own minds up, and that’s final. I mean [licence applicants] can appeal the decision in the Sheriff court, but, other than that, there’s no way to appeal to, to anyone…//.… So, not to sound big-headed in any way, but I don’t think there is a huge feeling of accountability from Board Members to anyone in particular.”

(LA1, LB Member)

This quotation also illustrates the relative lack of checks and balances that influence LBs (with the exception of appeals when these are launched by applicants). In their interview, the LB member quoted above was critical of this situation, perceiving that the LB can simply ignore national policy and makes decisions about the development of their Policy Statement unchecked.

### 3.6. Lack of Public-Administrative Accountability to Local Governance Hierarchy

Transitioning to an analysis of LBs’ public-administrative accountability within local-level governance, findings demonstrate Boards sit beyond local accountability regimes and lack local-level accountability.

“[The Board] has nothing to do with the other council structures. It’s a body on its own. It’s not accountable to anybody else in the council.”

(LA2, LB Member)

The above quotation is indicative of the consensus among interviewees that, at the organisation-level of public-administrative accountability, the LB is not accountable to the local council—it sits independently from the usual local accountability regime for local council committees.

Local and national level alcohol policy interviewees were critical of the lack of accountability of LBs to local governance bodies. In particular, there was recognition and frustration surrounding the inability of local public sector actors to hold LBs to account for work which affected other local organisations.

“Lots of activity at my level…working with the police and the [National Health Service], to try to influence the Licensing Board, nothing really happening, and then that frustration coming back at the Community Planning Partnership level because they can’t influence it either. So yeah, Licensing Boards, because of legislation, are sitting out here doing as they please without any accountability.”

(LA1, ADP Member)

Frustration was also expressed from national level stakeholders:

“Health is now one of the objectives of the licensing system, and health partners are statutory consultees. But that’s quite tricky…you’ve got locally elected members who sit on Licensing Boards, but the Licensing Board isn’t part of community planning…so you can have a Community Planning Partnership that say, alcohol’s a priority for us, and then you’ve got a local Licensing Board that basically says, who cares? You know, nothing to do with us, guv…so you’re ignoring the whole evidence base and there’s no accountability.”

(National Level 1)

This lack of accountability creates a barrier for effective achievement of alcohol policy goals, specifically the reduction of alcohol-related harm through the restriction of availability. The above quotation shows LBs can essentially ignore the goals and priorities of other local government entities, even when these priorities are directly related to LB decision-making (e.g., health as a licensing objective and alcohol problems as a local strategic priority). It also further shows LBs are able to make their own interpretations of existing public health evidence and have the autonomy to ignore it if they wish. This is problematic because it can create a tension between the LB and the broader policy context in which the national alcohol strategy and related local strategy planning have subscribed to this evidence.

### 3.7. Recent Changes to Licensing Board Accountability: Continued Need for Accountability Considerations

More recent legislation makes amendments to the existing regulatory regime surrounding LBs, which may have implications for their accountability. Specifically, relevant components of the *Air Weapons and Licensing Act 2015* (herein AWLA 2015) were developed following a 2012 consultation surrounding two main themes: Strengthening the powers of LBs and Police Scotland; and improving the effectiveness of the licensing regime [54]. It also introduced a mandate for LBs to report annually to the Scottish Government. Therefore, one might expect that accountability of LBs to the Scottish Government may change with more comprehensive enactment of the AWLA 2015.

A key component of the AWLA 2015 is the requirement of LBs to submit annual reports of their ‘functions,’ including a summary of decisions made by the LB, and a statement explaining how the LB has had regard to the licensing objectives [55]. In interviews, a Deputy Clerk for one LB was optimistic that this would enhance their accountability:

“You can have several months’ worth of [Licensing Board] business going by without much awareness, outside of what’s going on. And I think annual reporting, with specific information about the financial, you know, details of the annual fees coming in, details of what the fees are being used for, details of the numbers of applications, all of that. I mean, I don’t know, I think we’ll find out in due course, by way of regulation, what those annual reports will have to contain. But I think it’s a good thing.”

(Deputy Clerk)

The AWLA 2015 requires that the report must include statements regarding how the LB has had regard to the licensing objectives and their Policy Statement, as well as a summary about their decisions and the number of licenses in their area [55] (s.56 (2) (2)). However, although this section of the legislation was commenced in 2017, meaning the first reports were submitted in June 2018, the format and specific content of LB annual reports has not yet been published by the government [56].

Additionally, language in the legislation suggests that these requirements will have the same flexibilities as observed with other licensing legislation. For example, the AWLA 2015 states:

“A report under this section may include such other information about the exercise of the Licensing Board’s functions under this Act as the Board considers appropriate.”

(s.56 (2) (3), emphasis added)

This type of language suggests LBs will again be given significant autonomy in how they participate in this accountability reporting exercise, even regarding the type of information they wish to submit for scrutiny. Following Mark Bovens’ definition of accountability from the accountability literature [52,57], this type of statement, in which the potential ‘actor’ may use discretion to select what information they provide to the potential ‘forum’ (and thus what they have to explain/justify), undermines the possibility of establishing a robust accountability relationship between LBs and the Scottish Government.

Though the deputy clerk above was positive about this anticipated change, it was not always clear from interviews whether LB members were actually aware of this upcoming obligation. This indicates that the introduction of the annual reports was not communicated well to LBs, has not been prioritized by stakeholders in the licensing system, or both. This could further suggest that the stakeholders in the licensing system may not perceive the reports to be an important activity. For example, in LA3, multiple LB interviewees stated that they either had not studied the 2015 legislation yet or had only heard of it recently in a brief meeting discussion (despite these interviews having been conducted in late 2016). Overall, interviews with LB members indicated that the legislative changes were not yet implemented at the time of the interviews and did not suggest that there was any urgency to do so.

## 4. Discussion

The public health objective makes Scotland unique in its approach to licensing. However, the empirical results in this article suggested that LB members are not held sufficiently accountable for pursuing and protecting public health; a finding which emerged from analysis of the data. In particular, it was demonstrated that accountability mechanisms surrounding licensing do not currently allow for the objective to take its full effect. In starker terms, what has been observed is a tension between that which is written and suggested in the existing legal and policy framework surrounding alcohol licensing and that which is enacted in ongoing alcohol policy implementation practice, in terms of accountability mechanisms. The absence of a regular mechanism to ensure LBs are fulfilling their public health-related obligations means their decisions often present a challenge to alcohol policy implementation and the achievement of alcohol policy goals related to alcohol availability.

The concept of accountability was important here because it underpins how implementation occurs and is understood, as well as, importantly, who is (and how they are) responsible for undertaking implementation. The component parts of accountability processes—who is held responsible for policy implementation, how their actions are measured and judged, and how consequences are distributed—also provide insight into how governments perceive a given policy problem and its potential solutions. This article was concerned with the actions of public actors (i.e., those working in the public sector) who are often responsible for activities related to policy implementation [58]. What is known about accountability, and about the interplay of accountability with policy implementation processes, assisted the examination of how progress in Scottish alcohol policy implementation has been challenged or facilitated.

Thus, while the results presented in this article are aligned with existing research suggesting the public health objective has been difficult to implement [14,27], they also suggest that the lens of accountability is an important component of explaining why implementation has been challenging thus far. They lend support to an analysis which is focused on the accountability mechanisms surrounding licensing decision-making and its relationship with alcohol policy implementation.

### 4.1. Legislative and Legal Tensions

While a legal accountability system exists to regulate LBs, the arrangements are not conducive to protecting public health interests. As they stand, these arrangements do not adequately support the implementation of the public health objective (despite it being a component of the legal framework), creating a missed opportunity for supporting the implementation of the overall Scottish alcohol strategy.

To analyse this further, the data showed that public-administrative accountability of LBs relies on legal accountability arrangements; beyond legal accountability, there is a lack of other public-administrative mechanisms for holding LBs to account. This creates challenges for alcohol policy implementation and the pursuit of public health goals in this process, because any gaps or failures within legal accountability processes to support alcohol policy implementation cannot be mitigated by other public-administrative mechanisms. Indeed, while LB accountability is reliant on legal mechanisms, the practical arrangements of this are characterised by substantive flexibility and an imbalance towards wealthy industry stakeholders.

This leads to two observations. First, LBs’ flexibility to interpret legislation may allow them to contextualise the legislation (‘in light of their particular circumstances’); however, this also leaves them free to interpret the licensing objectives more flexibly than intended or to even ignore the ‘spirit’ of the legislation which seeks from LB members an understanding and concern about public health impacts of availability. This finding is aligned with results from a review of LB Policy Statements by Alcohol Focus Scotland, which showed that overprovision statements were varied in their breadth and strength [15], suggesting interpretative flexibility of availability-related evidence. This analysis also reaffirms research which finds that discretion among LBs can lead to inconsistent policy implementation [28].

Second, the observed imbalance towards wealthy licence applicants suggests LBs are only held to account by applicants who have the financial resources to challenge their decision-making on a legal basis. While the court case regarding MUP positioned the alcohol industry as an adversary of the government and its public health goals [59], the accountability structures and practices surrounding alcohol licensing continue to favour large industry retailers and largely exclude public health stakeholders. If this is the only mechanism that is effective for holding LBs to account, this excludes (a) alcohol policy implementers such as ADPs and their member organisations from holding LBs to account, because they cannot engage in the system in this manner, and (b) less financially secure licence applicants. The imbalance in this power distribution favours economic actors which also control significant financial power and resources, namely large industry producers and retailers. This creates a system in which powerful industry actors are also the actors who are most empowered to challenge the system that exists to regulate them. These findings are aligned with existing research in which public health actors perceive licensing processes as unfair, disempowering, and favouring of well-resources licensing actors [28]. Further, it is unlikely that this accountability mechanism will lead to the prioritisation of public health goals—it is located in the justice, not health, portfolio, and its imbalance towards large industry stakeholders means that the key interests of these stakeholders are also likely to be in conflict with public health objectives (i.e., industry is unlikely to trigger an appeal in pursuit of better public health-related outcomes). These findings are also aligned with a discussion of legal accountability in the broader public policy literature by Hill and Varone, where they highlight that the “law may be comparatively impotent in the face of complex issues of administrative discretion,” and that these concerns about the limits of legal control “stimulates a search for other models of accountability” (p. 344, [60]).

Thus, there is a tension in that, while licensing is meant to regulate alcohol retailing and availability, in practice the system still privileges large retailers. This problem is possibly perpetuated by the way the licensing system is currently meant to traverse multiple policy systems, i.e., justice/legal and public health, which have different approaches and priorities. This suggests that there is a need to effectively utilise systems of accountability to mitigate differences between these sectors, whereas, currently, they may be serving to perpetuate them.

Ultimately, the reliance on legal accountability of LBs and the characteristics of the enacted processes contribute to the tension between what is formally included in relevant alcohol licensing legislation and policy and what is observed in practice.

### 4.2. Democracy, Accountability, and Public Health in Scottish Alcohol Licensing

This analysis also highlights contextual and situational factors which further contribute to the identified tension. This included characteristics of local democracy, in particular in the roles of locally elected councillors and in the relationship between Scottish and local governments. The first example relates to the interplay of local democracy, accountability, and the economy. The data suggested that while LBs have a formal responsibility regarding the public health objective and no formal responsibility for being concerned with the economy, they often exhibited more concern for the latter. This could be a function of their simultaneous status as elected councillors and as LB members. As councillors, they have a responsibility for the economic wellbeing of their local area because they have been elected by their local community, and because the Scottish Government’s National Performance Framework demands that local government have regard to the government’s Purpose of economic growth (note, an updated 2018 version of the National Performance Framework states both ‘increased wellbeing’ and ‘economic growth’ as the foundational components of the government’s Purpose [17]). However, LB members legally have responsibility for public health, not economic concerns—the document guiding their decision-making states that commercial considerations are irrelevant to a policy which is designed to protect the wider public interest. Therefore, it is observed that their simultaneous roles of elected councillor and LB member seem to be in conflict, and that their democratic accountability and legal accountability are also in tension.

It is important to note that concerns regarding employment and the economy are relevant for health policy, particularly through a social determinants of health perspective [61,62]. It would be unwise to dismiss these concerns as irrelevant in this context—existing research has demonstrated, for example, that factors related to employment are associated with general health and mental health outcomes [63]. Interestingly, however, LB members did not frame their economic considerations in this way in interviews, even though this may have provided justification for their otherwise informal concerns. Instead they portrayed their concerns with employment and the economy as standalone and self-explanatory.

A second component of the analysis related to local democracy observes the differences between elected and non-elected alcohol policy implementation stakeholders. It was observed that councillors will experience democratic accountability which local, non-elected policy implementers (such as members of ADPs) will not. In light of this local political context there is a tension in how alcohol policy implementation is governed. Elected councillors are not part of the same systems of accountability as non-elected local government actors. However, the National Performance Framework identifies health as a Scottish priority, and LBs have a direct influence in contributing towards the alcohol-harm-related goals of the National Performance Framework because they control the availability of alcohol. It appears that their role as a LB member is again in tension with this councillor role, because being a LB member is a policy-led, administrative position which is explicitly intended to contribute to central government goals around alcohol-related harm [49], and one would presume that they should, as such, be held accountable for this policy work. Yet, this research identified no obvious mechanism through which LB members were held to account for this work. In light of these local contextual tensions and factors, local politics and councillors’ democratic accountability need to be acknowledged as important considerations underpinning LB accountability processes, with implications for alcohol policy implementation. This is a topic on which Fitzgerald and colleagues [28] have also made an important contribution, and this article complements that work by including non-public health interviewees (including LB members themselves) in a smaller number of local areas, allowing for an in-depth analysis specifically regarding public-administrative accountability surrounding LBs.

Third, LBs have a different arrangement of formal public-administrative accountabilities than other alcohol policy implementers, who are subject to more explicit accountability and reporting mechanisms within local and national governance systems. These differences are important because, as a consequence, LBs have greater discretionary powers and are subject to less oversight than other local policy implementers (e.g., ADPs). For example, LBs are quasi-judicial bodies which sit independently from the established accountability regime for other local council committees [64]. Thus, although LBs make decisions which influence the local populace and may impact local government progress towards their own strategic priorities, they sit beyond the accountability arrangements which could monitor them. This is potentially problematic because, if their actions present a barrier to the achievement of local strategies, local actors do not have any recourse to hold them to account for this. This demonstrates a key aspect of the LB accountability problem for alcohol policy implementation: While LBs sit beyond the system of public-administrative accountability applied to other alcohol policy implementers, they will continue to present a key challenge to achieving availability-related alcohol policy goals. If different stakeholders, who are meant to be allied and working towards shared policy goals, are not subject to similar accountability mechanisms, then there is a risk their actions diverge from one another, and possible come into conflict.

To illustrate this further, local authority areas have Local Development Plans which outline their visions and strategic priorities for their local communities [65]. These often include priorities around health and wellbeing. As a clear example of this, one region in Scotland, Aberdeenshire, has recently named ‘Changing Aberdeenshire’s Relationship with Alcohol’ as one of three local priorities for 2017-2027 [66]. In this context, implementation of a local strategy and the achievement of its intended alcohol-related outcomes will be challenged if the local LB acts with relative impunity to increase alcohol availability.

Finally, the data suggested that the Scottish Government is noticeably absent as an accountability forum for LBs, a situation of which many interviewees were critical. This current lack of accountability to the Scottish Government is problematic—if LBs are perceived as contributors to Scotland’s alcohol strategy, there must be a mechanism to ensure they act in this capacity, and the government is well-placed and legitimate to do this. However, it was noted that this would be complicated by the different levels of democracy at play, which influence LB members’ actions and have implications for whether the Scottish Government can hold them to account for their role in alcohol policy implementation. Despite this, the Scottish Government may be a well-positioned and legitimate organisation to shoulder both monitoring and accountability of LBs if this was arranged to be coherent with local autonomy and democratic accountability. Given the critique of the current situation by national and local alcohol policy stakeholders (including LB members) from a public health perspective, it will be important (and possibly timely) to push for system change.

### 4.3. Implications for Research and Policy

In light of the findings reported here and the knowledge gaps that remain, it is recommended that future research examine other aspects of accountability experienced by policy implementers. For example, this may include examining horizontal accountability(ies) to professional colleagues or a more nuanced analysis of perceptions of accountability to the public. Additionally, empirical analyses of how accountability regimes are aligned or conflict with other aspects of alcohol policy implementation processes will be needed if comprehensive understandings of implementation are to be attained.

Further, while existing alcohol policy implementation literature has included different considerations of accountability, there is a dearth of literature about alcohol policy which examines how, why, and in which circumstances different accountability mechanisms may be most effective. In terms of both research and policy implications, there therefore appears to be a need both to explore with stakeholders what alternative approaches to LB accountability may be effective and to develop a research agenda which investigates them empirically. As a first step, the findings reported here suggest introducing a monitoring system of LB licensing decisions (including whether objections were lodged by statutory consultees), which may provide useful data to inform future research and decision-making.

### 4.4. Strengths and Limitations

The combination in this study of interviews and document analysis permitted a timely, in-depth analysis of the interplay between accountability mechanisms and alcohol policy implementation in the context of Scottish alcohol licensing. However, local interviewees in this research were drawn from a subset of three local authority areas in Scotland; therefore, it is not possible to make broad generalisations nationally or internationally. Further, space did not permit discussion of other types of accountability which arose in the data as possibly impacting alcohol policy implementation—accountability within the LB itself or in relation to public involvement in licensing, for example.

## 5. Conclusions

In the context of limited empirical alcohol policy research which examines the interplay between alcohol policy implementation and accountability, this article makes a contribution to understanding how accountability processes influence the effectiveness of alcohol policy implementation in Scotland. In the case of Scottish alcohol licensing and the ‘public health objective,’ it was argued that there is a tension between the intentions of licensing legislation and the way it is enacted in practice. In particular, it suggests that there are a lack of accountability mechanisms acting upon Scottish LBs to ensure they contribute to the public health goals of the Scottish alcohol strategy. From a public health perspective, this has perpetuated a system in which LBs continue to act with problematic levels of flexibility and autonomy from the rest of the alcohol policy implementation system.

This article does not claim that Scotland’s alcohol strategy or, within licensing, public health objective itself has failed. Instead, it suggests that the implementation of these policies is suffering from challenges that are well-known in the wider policy implementation and governance literature: That it is insufficient to develop public health policy or legislation and expect that the implementation of this will straightforwardly follow from this top-down decision. In this case, this problem is particularly acute given the complex interplay of public health, economic, democratic, and governance concerns which influence the decisions and actions of alcohol policy implementation stakeholders. The key message, however, is that national alcohol policy in Scotland will fall short of intended goals as long as the tension between licensing legislation and enacted licensing practice remains.

## Figures and Tables

**Table 1:** Membership and Responsibilities of Scottish Alcohol Policy Implementation Stakeholders.

	Membership	Responsibilities Regarding Alcohol Policy Implementation
Scottish Government	Elected Ministers and unelected civil servants	Designated government teams are responsible for developing national alcohol policy and supporting its implementation.
Licensing Boards	Elected Local Councillors	Boards preside over the local alcohol licensing system, which controls alcohol availability [18].
Alcohol and Drug Partnerships	Range of statutory (e.g., health, police, social work, education, fire service) and non-statutory (e.g., third sector) representatives	These partnerships are tasked with local alcohol policy implementation. ADPs develop local alcohol strategies which serve to translate and tailor the national alcohol strategy to local needs.

**Table 2:** Legislative provisions regarding Licensing Board accountability.

Legislation	Relevant Provisions Regarding Accountability of Licensing Boards
Licensing (Scotland) Act 2005	Mandates that the Board produce a Licensing Policy Statement once every three years, which provides a locally-specific legal basis for their decision-making. Mandates that the Licensing Statement include statement on whether local areas are overprovided for (enacted 2009). Outlines the five licensing objectives, including protecting and improving public health.
Alcohol etc. (Scotland) Act 2010	Makes modifications to mandatory conditions of premises and occasional licences which were in the 2005 Act. Sets out actions Licensing Board must undertake before and after it makes a variation to premises licence conditions. It also states that a variation to licence conditions may be made only where the Board is satisfied that the variation is necessary or expedient for the purposes of any of the licensing objectives. Amends the 2005 Act to add the relevant Health Board to the bodies that the Licensing Board is required to consult when developing their Licensing Policy Statement, a Health Board which must also be notified of any premises licence applications.
Criminal Justice and Licensing (Scotland) Act 2010	Makes modifications to the 2005 Act regarding application notification requirements, occasional licenses, hours, etc.
Air Weapons and Licensing (Scotland) Act 2014	Mandates Licensing Boards submit annual reports of functions to the Scottish Government, which must include a statement explaining how the Board has had regard to the licensing objectives and their Licensing Policy Statement when carrying out its functions.

**Table 3:** Number of local- and national-level interviewees.

Sector	Number of Interviewees by Local Area and Nationally		
	LA1	LA2	LA3
Licensing Board Members	4	1	3
Other local alcohol policy implementers (e.g., ADP members)	13	15	18
National Level Alcohol Policy Stakeholders	9		
